# Atmospheric Gas-Phase Catalyst Etching of SiO_2_ for Deep Microfabrication Using HF Gas and Patterned Photoresist

**DOI:** 10.1021/acsami.4c01291

**Published:** 2024-04-23

**Authors:** Ko-hei Sano, Yoshitaka Ono, Ryosuke Tobinaga, Yutaka Imamura, Yasuo Hayashi, Takahiko Yanagitani

**Affiliations:** † Graduate School of Advanced Science and Engineering, Waseda University, Tokyo 169-8555, Japan; ‡ Innovative Technology Laboratories, AGC Incorporated, Kanagawa 230-0045, Japan; § Kagami Memorial Research Institute for Material Science and Technology, Waseda University, Tokyo 169-0051, Japan; ∥ JST CREST, Saitama 332-0012, Japan; ⊥ JST FOREST, Saitama 332-0012, Japan

**Keywords:** gas-phase catalyst etching, deep microfabrication, SiO_2_, HF gas, organocatalyst, smooth-sidewall surface

## Abstract

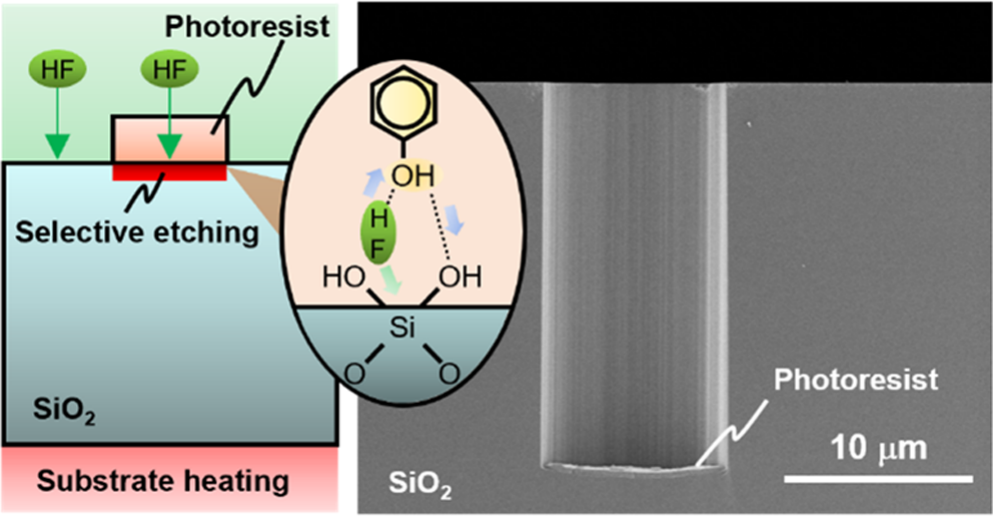

Micro/nanoscale structure fabrication is an important process for designing miniaturized devices. Recently, three-dimensional (3D) integrated circuits using SiO_2_ via-holes interlayer filling by copper have attracted attention to extend the lifetime of Moore’s law. However, the fabrication of vertical and smooth-sidewall via-hole structures on SiO_2_ has not been achieved using the conventional dry etching method due to the limitation of the selective etching ratio of SiO_2_ and hard mask materials. In this study, we developed a unique method for the deep anisotropic dry etching of SiO_2_ using atmospheric gas-phase HF and a patterned photoresist. The hydroxyl groups in the photoresist catalyzed the HF gas-phase dry etching of SiO_2_ at high-temperature conditions. Therefore, fabrication of vertical with smooth-sidewall deep microstructures was demonstrated in the photoresist-covered area on SiO_2_ at a processing rate of 1.3 μm/min, which is 2–3 times faster than the conventional dry etching method. Additionally, the chemical reaction pathway in the photoresist-covered area on SiO_2_ with HF gas was revealed via density functional theory (DFT) calculations. This simple and high-speed microfabrication process will expand the commercial application scope of next-generation microfabricated SiO_2_-based devices.

## Introduction

The realization of a microfabrication process for the construction of high-aspect-ratio micro/nanoscale structures on SiO_2_ has been a long-standing goal for the development of novel devices in nanotechnology. The “Bosch process”—a deep microfabrication method for Si invented by Robert Bosch GmbH in 1992^1^—is widely used to fabricate advanced Si-based devices such as micro-electromechanical systems^2,3^ and through-silicon vias.^4,5^ In contrast to pure Si, SiO_2_ exhibits high chemical resistance, high transparency in the visible range, low thermal conductivity, and extremely low dielectric loss at high frequencies.^6,7^ By exploiting these unique characteristics, various SiO_2_-based devices that require vertical microstructures with smooth sidewalls have been developed in recent years, including microfluidic chips^8,9^ and optical meta-surfaces.^10,11^ Anisotropic microstructures on SiO_2_ are typically fabricated via inductively coupled plasma reactive ion etching (ICP-RIE).^12−14^ However, the fabrication of high-aspect-ratio vertical and smooth-sidewall microstructures has not been achieved using this method because hard mask materials are etched via ion irradiation from an ICP source.

Surface treatment with an anhydrous HF gas is a plasma-free dry etching method for SiO_2_.^15−17^ The chemical reaction of SiO_2_ with HF gas can be expressed as follows:
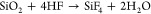
In general, the surface treatment of SiO_2_ with HF gas is not suitable for anisotropic etching because gas-phase chemical reactions proceed isotropically on SiO_2_. In addition, the dry etching reaction of SiO_2_ with HF gas is extremely slow even at high temperatures of >600 °C.^17^ However, the chemical reaction of SiO_2_ can be accelerated by the formation of hydrogen bonds between the HF gas and H_2_O molecules.^15,16,18,19^ We hypothesized that hydrophilic functional groups in organic materials, such as R–OH or R–NH_2_, also accelerate the dry etching reaction because these functional groups can form hydrogen bonds with the HF gas. A novolac-type photoresist, which contains hydroxyl groups, was examined in this study. The photoresist allows high-resolution fine patterns to be easily formed using conventional lithography. Therefore, we consider that by employing the photoresist on the SiO_2_ as the dry etching accelerator, the deep microfabrication of SiO_2_ can be achieved through surface treatment with HF gas. Similar concepts of microfabrication process, metal-assisted chemical etching (MacEtch) of Si using noble metals as an etching accelerator and HF/H_2_O_2_ solution, have attracted attention in recent years.^20−24^ The noble-metal-covered area on Si is actively oxidized by the H_2_O_2_. Thus, the Si in the oxidized area is selectively removed by the HF. According to this mechanism, stable oxide materials such as SiO_2_ are not suitable for selective etching via the MacEtch process.

The chemical reactions of photoresists on SiO_2_ under HF gas treatments have been investigated. The earliest report, which was published in 1977, introduced the “DryOX” method,^25^ wherein a thin oxide film on a Si wafer covered by a negative-tone photoresist was selectively removed using HF gas in vacuum conditions. The interaction of HF molecules with the carbonyl groups of the photosensitizer in the negative photoresist promotes the formation of HF_2_^–^.^26^ Selective etching of the thin oxide film under the photoresist was realized by using HF_2_^–^ species. A deep SiO_2_ etching method involving a low-temperature HF gas treatment in vacuum conditions and the use of a photoresist was reported in 2003.^27^ This process actively used H_2_O generated from the reaction between SiO_2_ and the HF gas. The defining characteristic of this process is the cooling of the substrate below 20 °C during the HF gas treatment, which enhances the generation of HF_2_^–^ produced by the reaction of H_2_O and HF in the photoresist-covered area on SiO_2_. Therefore, selective etching under the photoresist was performed previously through the formation of HF_2_^–^, which are employed as active species in the conventional wet-type etching of SiO_2_ using liquid HF.^28^ However, the gas-phase HF_2_^–^ or H_2_O molecules likely adsorbed on the etched sidewall surface during this process, accelerating the side-etching reaction at processing temperatures below 100 °C.

In this study, we develop a novel method for high-temperature atmospheric HF gas-phase deep etching of SiO_2_ using a novolac-type photoresist as a catalyst. The acceleration of the etching reaction of H_2_O molecules was prevented by employing processing temperatures of >100 °C. Additionally, a deep trench structure with a vertical and smooth sidewall is obtained as the side-etching reactions are suppressed because selective etching occurs only in the catalyst contact area on SiO_2_ without producing HF_2_^–^. Furthermore, high-speed anisotropic etching is realized because the increased process temperature accelerates the chemical reaction on the SiO_2_ substrate. Our simple method requires no plasma source, vacuum chamber, or hard mask materials, which are indispensable in conventional processes. Moreover, we examine the chemical reaction pathway in the photoresist-covered area on SiO_2_ with HF gas using density functional theory (DFT) calculations.

## Results and Discussion

### HF Gas-Phase Anisotropic Etching Using a Photoresist

**Figure 1:**
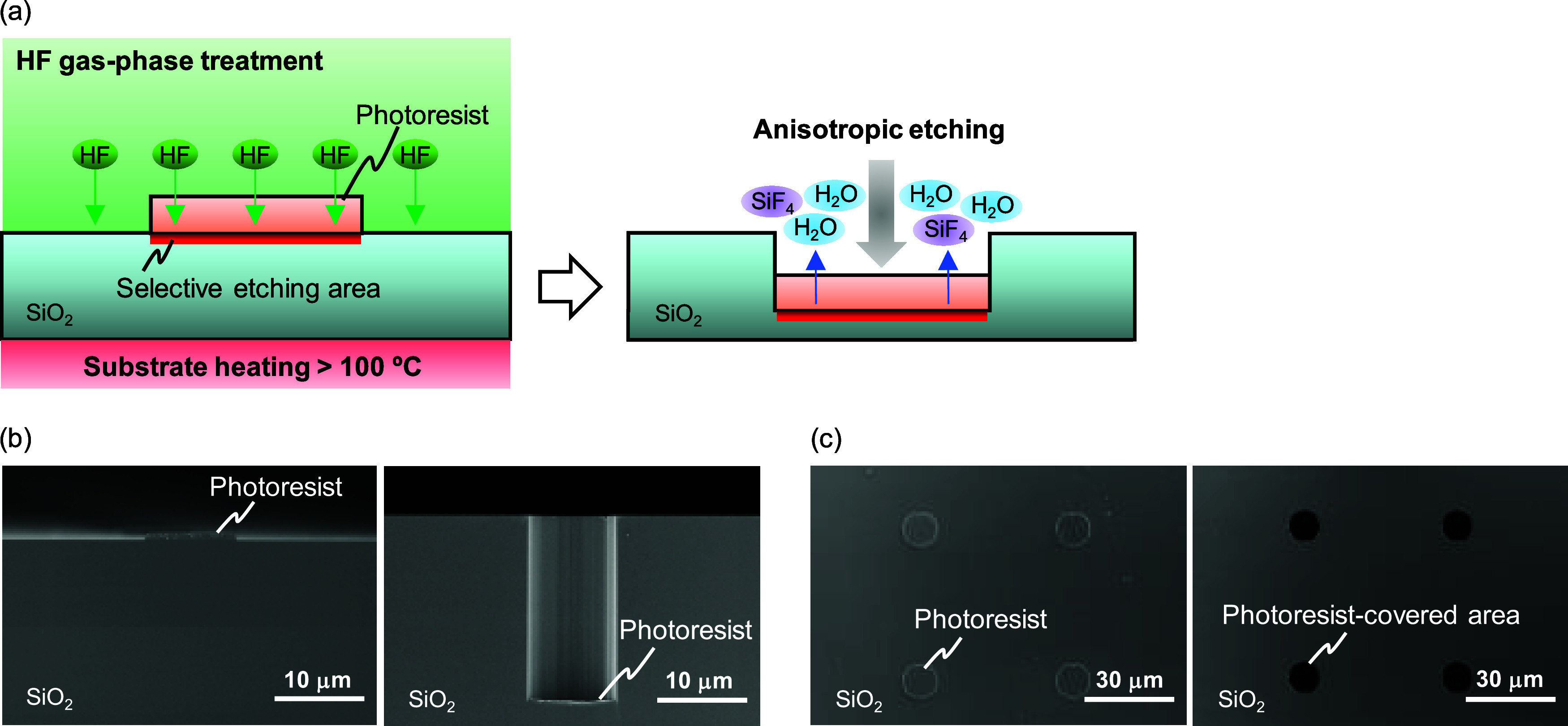
HF gas-phase anisotropic etching of silica glass substrates in photoresist-covered areas. (a) Concept of HF gas-phase anisotropic etching during processing (left) and after HF gas-phase treatments (right). (b) Cross-sectional scanning electron microscopy (SEM) images of the photoresist on SiO_2_ before (left) and after (right) HF gas-phase treatment. (c) Top surface SEM images of photoresists on SiO_2_ before (left) and after (right) HF gas-phase treatment.

The hydrophilic functional groups in the photoresist accelerate the chemical reaction between HF gas and SiO_2_. In addition, the side-etching reaction is suppressed by high-temperature conditions at >100 °C because H_2_O molecules, which serve as the reaction accelerator, cannot be adsorbed onto the substrate. Accordingly, vertical deep microstructures can be fabricated via selective etching due to the HF gas that passes through the photoresist on SiO_2_, as shown in Figure 1a. To verify this hypothesis, HF gas-phase surface treatment was performed at 250 °C for 1000 s using a sample with a patterned photoresist on a silica glass substrate. A vertical deep hole structure with a smooth-sidewall surface was formed in the photoresist-covered area, as shown in Figure 1b. Additionally, the photoresist was observed at the bottom of the hole structure, indicating that the deep microstructure was fabricated in a photoresist-covered area via selective etching with HF gas. Conversely, no structural changes were observed in the areas of bare SiO_2_ in the sample after the surface treatment, as shown in Figure 1c.

**Figure 2:**
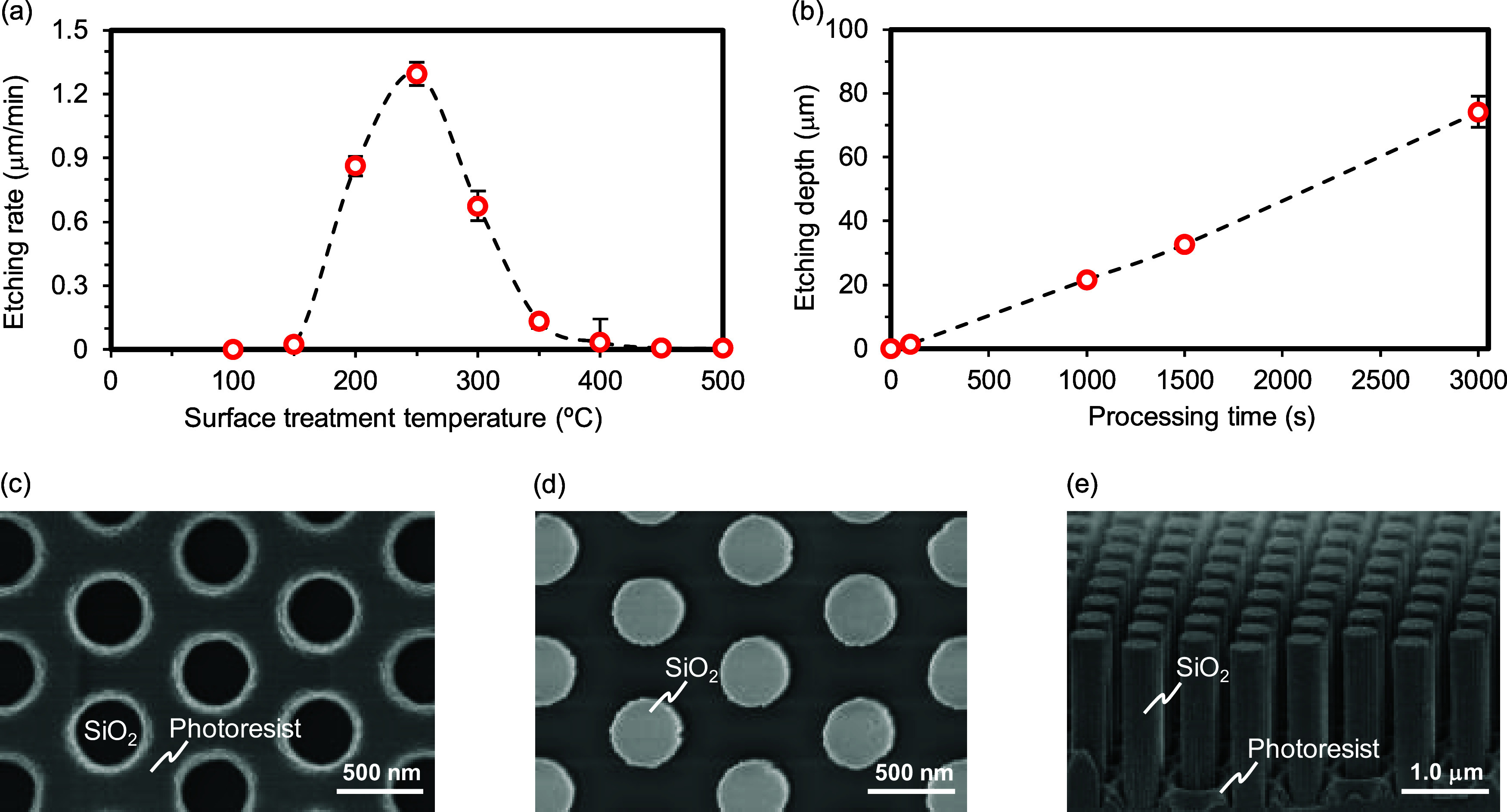
Characteristics of HF gas-phase anisotropic etching for silica glass substrates in photoresist-covered areas. (a) Etching rates at different processing temperatures. The error bars show the 3σ standard deviations (*n* = 5). (b) Etching depth under 250 °C surface treatment for different processing times. The error bars show the 3σ standard deviations (*n* = 5). (c) Top surface SEM image of photoresists with submicrometer hole patterns on SiO_2_. (d) Top surface SEM image of nanopillar arrays on SiO_2_ fabricated via HF gas-phase surface treatment using a photoresist with submicron hole patterns. (e) 30°-tilted SEM image of nanopillar arrays on SiO_2_ fabricated via HF gas-phase surface treatment with submicron hole patterns.

The anisotropic etching rates in the photoresist-covered area at different processing temperatures are shown in Figure 2a. The dry etching rate increased sharply from 100 to 200 °C and reached a maximum at 250 °C. The maximum dry etching rate (∼1.3 μm/min) was approximately 2–3 times faster than that of conventional methods such as ICP-RIE.^12−14^

The dry etching depth for each processing time under a surface treatment at 250 °C is plotted in Figure 2b. The etching depth was almost linear with respect to the processing time and reached 76 μm when the surface treatment was performed for 3000 s. This indicated that the etching reaction continued when sufficient HF gas was supplied to the photoresist at the bottom of the trench. Gas-phase molecules can generally diffuse easily through micrometer-scale gaps. Therefore, this HF gas-phase anisotropic etching allows for deep microfabrication without depth limitations.

The resolution of the HF gas-phase anisotropic etching depends on the photoresist patterns on the substrate. In particular, the novolac-type photoresist can be fabricated with a submicrometer-scale pattern in a large area using a general lithography system. Submicron-scale structures with high aspect ratios, such as nanopillar arrays, are highly demanded for biological applications^29−31^ or optical devices.^10,11,32^ A novolac-type photoresist with submicrometer hole patterns was fabricated on SiO_2_, as shown in Figure 2c. The photoresist-covered areas were selectively etched via the HF gas-phase surface treatment, as shown in Figure 2d. Additionally, vertical nanopillar arrays with the same diameter as the photoresist holes were fabricated on SiO_2_, as shown in Figure 2e. Therefore, deep microfabrication at the submicron-scale structure was demonstrated using the HF gas-phase anisotropic etching technique with a novolac-type photoresist. In addition, the vertical nanopillar structure with the same diameter as that of the catalyst pattern indicates that the effect of lateral etching on the sidewalls is negligible in this process.

### Anisotropic Dry Etching Rate Reduction Using Thermally Decomposed Photoresists

**Figure 3:**
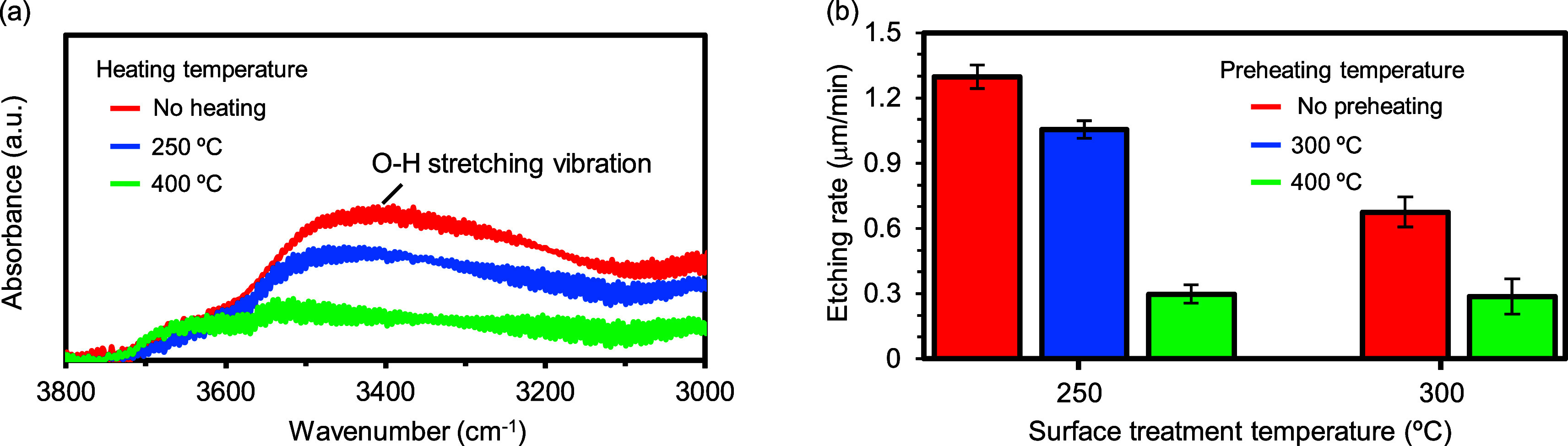
Thermal decomposition of novolac-type photoresists. (a) O–H stretching vibrations in the FT-IR spectra of the heated photoresists. (b) HF gas-phase anisotropic etching rate of silica glass substrates at 250 and 300 °C using preheated photoresists. The error bars show the 3σ standard deviations (*n* = 5).

The HF gas-phase anisotropic etching method developed in this study involves a chemical reaction between HF gas and photoresist on the SiO_2_ substrate. The gas-phase chemical reaction is typically accelerated by increasing the processing temperature. However, the etching rate was maximized at a processing temperature of 250 °C. We considered that the photoresist was thermally decomposed at high-temperature conditions. We hypothesized that the hydroxyl groups in the photoresist played two roles in the etching process: catalytic function on the etched surface and incorporation of HF molecules into the photoresist. Therefore, the etching reaction was suppressed because of the reduced number of hydroxyl groups in the photoresist at processing temperatures of >250 °C. To verify the reduction in the etching rate due to the thermal decomposition of the photoresist, the hydroxyl groups in the heated photoresist were analyzed via Fourier transform infrared (FT-IR) spectroscopy, as shown in Figure 3a. The intensity of the O–H stretching vibration peaks in the spectrum of the photoresist decreased with an increase in temperature, confirming that the number of hydroxyl groups in the photoresist decreased upon heat treatment. Samples with fewer hydroxyl groups in the photoresist after the heat treatment were subjected to the HF gas-phase surface treatment, as shown in Figure 3b. The etching rates of the preheated samples were significantly reduced at processing temperatures of 250 and 300 °C owing to the fewer hydroxyl groups in the photoresist. Thus, 250 °C is considered the optimal processing temperature for the HF gas-phase anisotropic etching as it accelerates the chemical reaction and suppresses the thermal decomposition of the photoresist.

### Characteristics of Microstructures Fabricated via HF Gas-Phase Anisotropic Etching

The processing rate of the HF gas-phase anisotropic etching of SiO_2_ was higher than that achieved by the ICP-RIE process. The verticality and smoothness of the sidewall surface in the microstructures are critical requirements for the microfabrication techniques. Thus, the taper angles and roughness of the sidewalls of the trench structures fabricated via HF gas-phase anisotropic etching and ICP-RIE were evaluated. Table 1 presents the taper angles and arithmetic average roughness (*R*_a_) values of the sidewalls obtained by using different microfabrication methods under various conditions. The microstructures fabricated via the HF gas-phase anisotropic etching exhibited vertical and smooth-sidewall structures, even at etching depths of >10 μm. These excellent shapes can be easily fabricated via the HF gas-phase anisotropic etching because side-etching reactions are suppressed by the high temperature, and the shape of the photoresist does not change during the chemical reaction. The trench structures fabricated via ICP-RIE using Cr hard masks with thicknesses of 0.2 and 1.0 μm exhibited taper angles of 9.7 and 2.0°, respectively. In addition, the trench structure with a larger taper angle exhibited a smoother sidewall surface. The ion irradiation of the sidewall surface occurs when the sample has a large taper angle. Therefore, a sidewall surface with a large *R*_a_ value is smoothened via ion irradiation. Thus, the fabrication of vertical and smooth-sidewall microstructures on SiO_2_ was difficult using the ICP-RIE method. In addition, the bottom surface roughness of the microstructure fabricated via HF gas-phase anisotropic etching at 250 °C was evaluated after photoresist stripping. The *R*_a_ value of the bottom surface was 1.2 nm, which was lower than that of the sidewall surface. Therefore, a vertical and deep microstructure with smooth side and bottom surfaces was obtained on the SiO_2_ substrate via HF gas-phase anisotropic etching.

**Table 1:** Etching Depths, Taper Angles, and *R*_a_ Values of the Sidewall of Trench Structures Obtained by Using HF Gas-Phase Anisotropic Etching and ICP-RIE under Different Conditions

microfabrication method	etching depth (μm)	taper angle (deg)	*R*_a_ of sidewall (nm)
HF gas-phase anisotropic etching (250 °C)	6.3	0.9	3.0
HF gas-phase anisotropic etching (250 °C)	12.0	0.9	4.1
HF gas-phase anisotropic etching (350 °C)	3.8	0.1	2.3
HF gas-phase anisotropic etching (350 °C)	7.8	0.3	2.8
ICP-RIE (Cr hard mask: 0.2 μm)	6.6	9.7	6.0
ICP-RIE (Cr hard mask: 1.0 μm)	7.0	2.0	15.8

**Figure 4:**
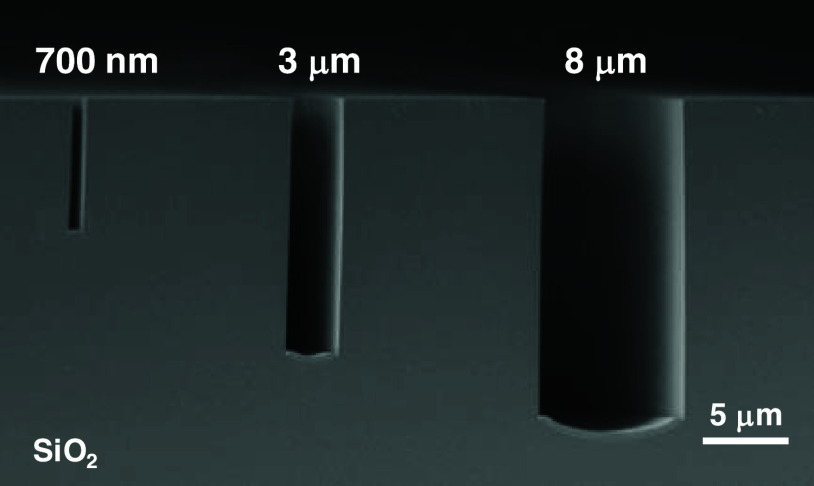
Cross-sectional SEM image of the SiO_2_ substrate microfabricated via HF gas-phase anisotropic etching. The surface treatment was performed at 300 °C for 2000 s using a novolac-type photoresist with different widths as a catalyst.

Aspect-ratio-dependent etching (ARDE)^33−36^ is a serious problem in the microfabrication using the RIE process. The linear trench structures of different widths on the same SiO_2_ substrate were fabricated via HF gas-phase anisotropic etching, as shown in Figure 4. The etching depth is ranked in the order 8 μm > 3 μm > 700 nm, which is a similar trend to the ARDE. We considered that the differences of etching rate occurred due to the thermal decomposition of the photoresist. Thermal decomposed reaction in the photoresist is thought to proceed from the surface layer. In this case, the narrower width photoresist was a high percentage of the thermal decomposed volume because of their large specific surface area. Therefore, the ARDE of the HF gas-phase anisotropic etching occurred due to the differences of the amount of hydroxyl groups on the SiO_2_ surface between the narrow and wide-width photoresists.

### Mechanisms of HF Gas-Phase Anisotropic Etching of SiO_2_ using Novolac-Type Photoresists

We examined the mechanism of the HF gas-phase anisotropic etching of SiO_2_ via the generation of gas-phase SiF_4_ and H_2_O. According to the previously reported models in which the reaction of SiO_2_ with HF was studied using computational simulations,^18,19,37−40^ the reaction pathway of SiO_2_ with HF gas using the novolac-type photoresists was investigated via DFT calculations.

**Figure 5:**
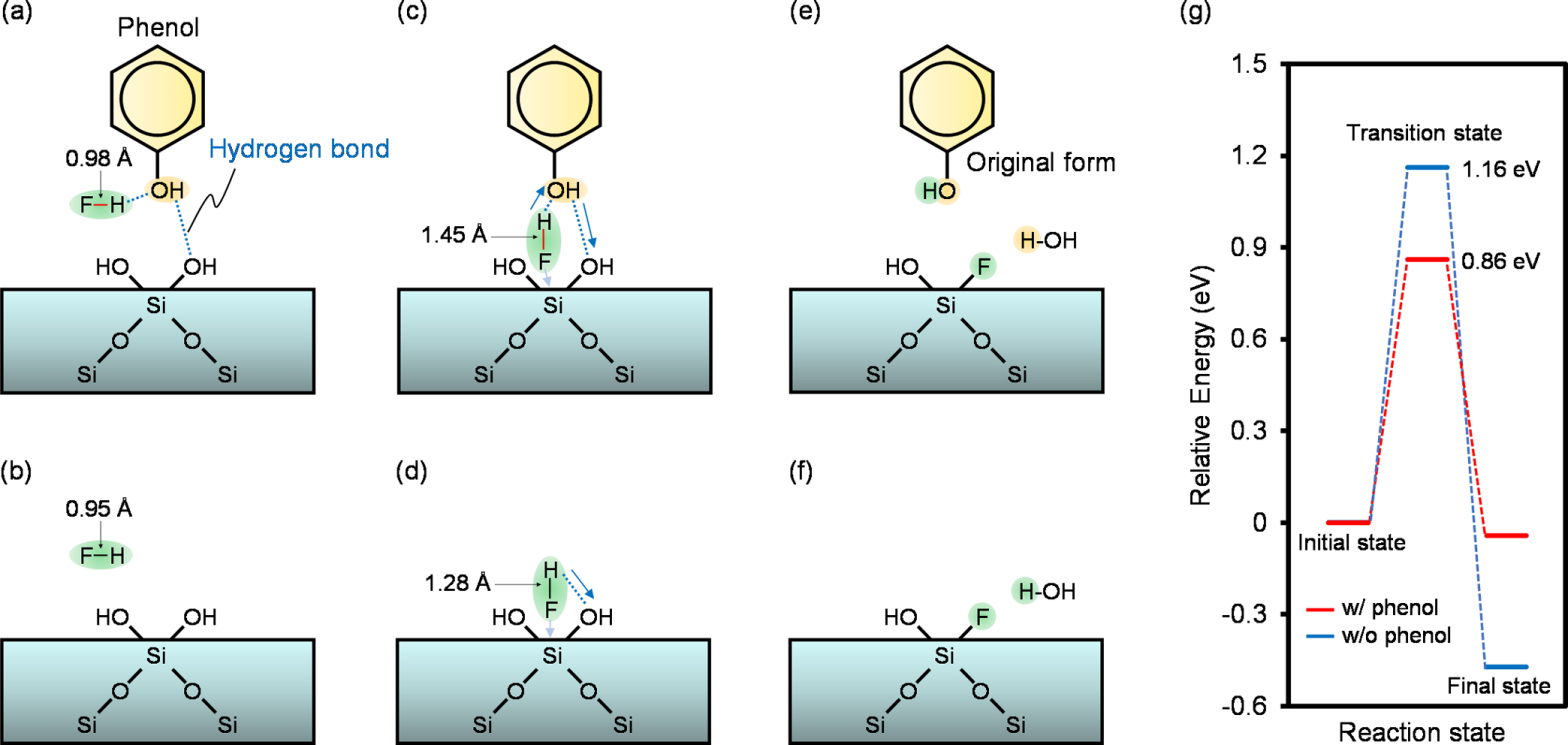
Chemical reaction pathway of the HF gas-phase etching of SiO_2_. (a) Initial state of SiO_2_ dry etching in the presence of phenol. (b) Initial state of SiO_2_ dry etching in the absence of phenol. (c) Transition state of dry etching when the HF molecule approaches the Si atom in the presence of phenol. (d) Transition state of dry etching in the absence of phenol. (e) Final state after replacement of Si–OH with a F atom on SiO_2_ when a phenol interacts with SiO_2_. (f) Final state after replacement of Si–OH with a F atom on SiO_2_ in the absence of phenol. (g) Relative energy diagram (total energy of the initial state in each pathway set to zero) from the initial state to the final state along the transition state in the presence of phenol and in the absence of phenol.

The initial state of SiO_2_ and HF with phenol, which is the catalytic group of the novolac-type photoresist, is shown in Figure 5a. The initial state was carefully chosen so as to be the most stable among possible geometries. The hydroxyl group of phenol interacts with HF and Si–OH, which increases the H–F bond length by 0.03 Å relative to that in the initial state in the absence of phenol (Figure 5b), indicating that the H–F bond is weakened by the interaction. The F atom in the HF molecule attacks the Si atom on the SiO_2_ surface, which leads to the transition state, as shown in Figure 5c. The H–F bond length in the transition state is increased by 0.17 Å, relative to that in the transition state in the absence of phenol, as shown in Figure 5d. Therefore, the nucleophilic substitution of the F atom with the Si atom proceeds readily in the presence of phenol because the aforementioned interaction with the phenol weakens the H–F bond in the transition state. Finally, the hydroxyl group on SiO_2_ is replaced by the F atom via nucleophilic substitution (Figure 5e), after which the H atom of the HF molecule is transferred to the hydroxyl group of the phenol. Additionally, an H_2_O molecule is generated by the reaction of Si–OH with a H atom derived from the phenol. Subsequently, the hydroxyl group of the phenol is restored via that proton transfer mechanism. The energy barrier to Si–F bond formation in this reaction pathway is calculated to be 0.86 eV, which is 0.30 eV lower than that in the reaction pathway without phenol (Figure 5f), as shown in Figure 5g. These results indicate that the novolac-type photoresist catalyzes the dry etching of SiO_2_ using HF gas.

**Figure 6:**
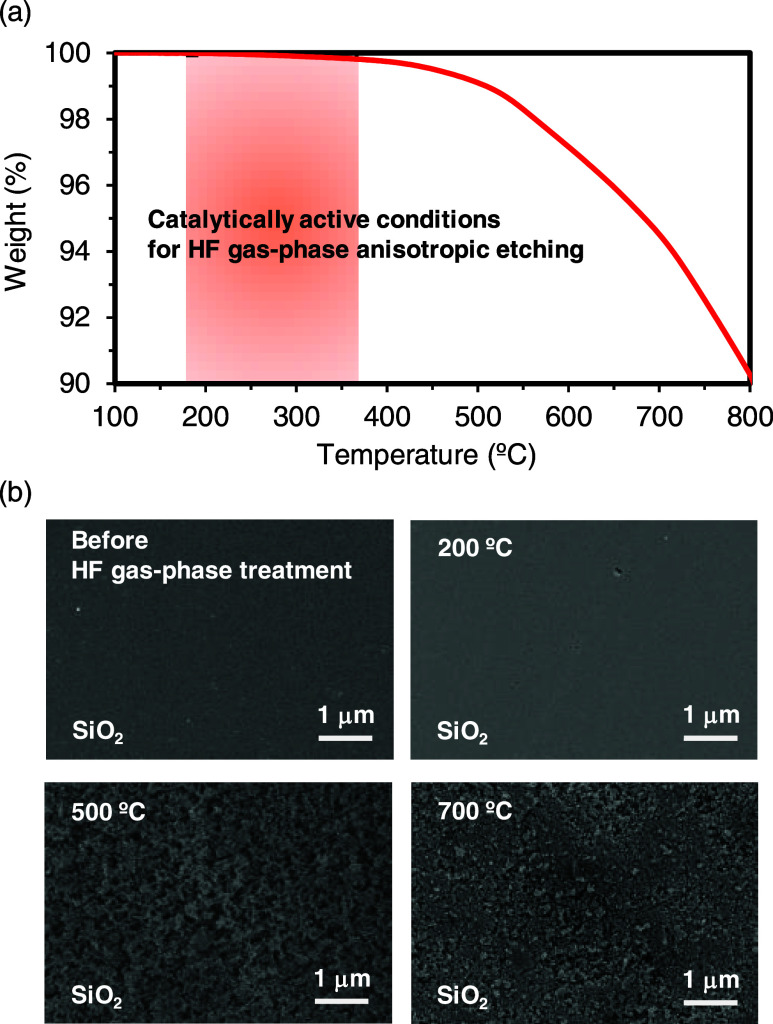
Characteristics of HF gas-phase dry etching of SiO_2_ in the absence of catalysts. (a) TGA curve of SiO_2_ in HF gas-phase condition. (b) Top surface SEM images of SiO_2_ after HF gas-phase treatment at high-temperature conditions.

The dry etching characteristics of SiO_2_ in the absence of catalysts were then experimentally investigated by using thermogravimetry analysis (TGA) under HF gas-phase conditions, as shown in Figure 6a. The weight loss due to the etching reaction in the temperature range of 100–300 °C was less than 0.1%. On the other hand, significant weight loss was observed when the treatment temperature exceeded 400 °C. The SiO_2_ surface after HF gas-phase treatment at each processing temperature without catalysts is shown in Figure 6b. A smooth surface was observed under the 200 °C condition. However, surfaces with large roughness due to the etching reactions were observed at above the 500 °C conditions. These results indicate that high-temperature conditions above 400 °C are required for the dry etching of pure SiO_2_ with HF gas. Therefore, the etching reaction on the SiO_2_ surface not covered by the photoresist and etched sidewalls did not proceed under the catalytically active conditions because of the high reaction barrier energy in the catalyst-free reaction pathway. Thus, the vertical microstructures with smooth-sidewall surfaces were fabricated without using hard mask materials because of the excellent catalytic activity of hydroxyl groups in the novolac-type photoresist.

## Conclusions

Inspired by the chemical reaction of HF gas-phase surface treatment in the presence of H_2_O molecules, deep anisotropic dry etching of SiO_2_ was realized under atmospheric conditions by utilizing the hydroxyl groups in a novolac-type photoresist as catalysts. The method imparts catalytic functions to the photoresist-covered area on the substrate at high temperatures, which accelerates anisotropic etching and prevents the adsorption of H_2_O molecules on the etched sidewall. Therefore, vertical and smooth-sidewall microstructures were easily fabricated on the SiO_2_ at the processing rate of 1.3 μm/min without using plasma sources, a vacuum chamber, or hard mask materials. In addition, fabrication of nanopillar arrays which have critical roles in next-generation biological or optical applications was demonstrated. This process enables the submicron-scale deep microfabrication without depth limitations. Therefore, microstructures with a high aspect ratio of 100 or more can be formed on the SiO_2_ substrate via HF gas-phase anisotropic etching. Moreover, according to the anisotropic etching mechanisms established in this study, this process may be applicable to materials other than SiO_2_ (such as SiN or SiC). This simple atmospheric gas-phase catalyst etching process may facilitate the development of new-conceptual microfabricated applications in various fields.

## Methods

### Sample Preparation

Optical-grade synthetic silica glass substrates (square; length: 25 mm; thickness: 0.5 mm) were cleaned via ultraviolet (UV) ozone treatment for 15 min, treated with hexamethyldisilazane, and coated with photoresists. Positive-tone novolac-type photoresists (THMR-iP 3100, Tokyo Ohka Kogyo, Japan) were spin-coated on the substrate at 2500 rpm for 25 s. Periodic 10-μm-diameter dots were patterned on the substrate over an area of 20 mm^2^ using a maskless photolithography system (DL-1000A2, Nano System Solutions, Japan) and an alkaline developer solution (NMD-3, Tokyo Ohka Kogyo, Japan). Finally, organic contaminants were removed from the parts of the substrate surface that were not covered by the photoresist pattern via UV-ozone cleaning.

### Surface Treatment with HF Gas

The high-temperature HF gas-phase surface treatment of the samples was performed using a rapid thermal annealing system (VHC-P610, ULVAC, Japan). The etched samples were then placed on a carbon plate heated to >100 °C via IR heating. The gas-phase conditions of the samples were controlled via local gas injection. The chamber of the rapid thermal annealing system was evacuated to be <20 Pa using a rotary pump and subsequently returned to the atmospheric pressure by supplying dry N_2_ gas. The amount of H_2_O in the chamber was reduced by replacing the air with low-dew-point N_2_ gas. N_2_ gas was then passed through the exhaust valve at a rate of 5.0 standard liters per minute (SLM) under atmospheric conditions. The sample was then heated under surface treatment conditions for 2 min, and the temperature was maintained for 1 min to stabilize the sample surface temperature. Finally, HF gas-phase surface treatment of the sample was performed by switching the gas line in front of the local injection tube. The HF and N_2_ gases were used as the surface treatment gases at flow rates of 1.0 and 4.0 SLM, respectively.

Characteristics of the HF gas-phase dry etching of SiO_2_ in the absence of catalysts were investigated using a thermogravimetric analyzer (STA2500 Regulus, NETZSCH, Germany). An alumina container with 50 mg of SiO_2_ powder was used as the etching sample. The amount of H_2_O and organic contaminants in the samples was reduced by annealing to 500 °C under N_2_ conditions before the etching tests. The temperature conditions were increased from 100 to 800 °C at 10 °C/min. The gas-phase condition was HF/N_2_ = 10 vol% flowing at 50 standard cubic centimeters per minute under atmospheric pressure conditions.

### Anisotropic Dry Etching with ICP-RIE

Samples fabricated via the ICP-RIE method were prepared by using two types of Cr films with thicknesses of 0.2 and 1.0 μm deposited on the silica glass substrate, which served as the hard mask material for the RIE. The Cr films were patterned into linear shapes using a wet-type chemical etching liquid (Cr201, Kanto Kagaku, Japan). The trench structure at a depth of 6–7 μm was fabricated using ICP-RIE with CHF_3_ and CF_4_ gases.

### Characterization

The etched structures were examined via field-emission SEM (SU8000, Hitachi, Japan). The *R*_a_ of the etched sidewall was measured by using inline three-dimensional (3D) atomic force microscopy (NX-3DM, Park Systems, Korea). The functional groups in the photoresist were analyzed via FT-IR spectroscopy (NICOLET iS50 FT-IR, Thermo Fisher Scientific) to investigate the thermal decomposition that occurred at various processing temperatures with the photoresist on a silica glass substrate, which was heated at each temperature for 2 min.

### DFT Calculations

The mechanism of HF gas-phase anisotropic etching of SiO_2_ using the novolac-type photoresist was investigated using DFT calculations at the B3LYP^41,42^/6-311 + G(d, p) level of theory using the Gaussian 16 program package.^43^ The default conditions for all geometric optimization calculations were adopted.^44^ In the DFT model, a β-cristobalite (100) cluster structure with dangling Si bonds terminated with −OH was used as an initial geometry instead of silica glass. A cluster model was used for DFT calculations of amorphous solids such as glasses in previous studies.^18,37^ This cluster structure was fully geometry optimized and exhibited no imaginary vibrations during vibrational analysis. The novolac-type photoresist was modeled as a single phenol molecule that plays a key catalytic role. In the first step of the reaction pathway calculation, the phenol interacted with both an HF molecule and Si–OH in an initial structure. The interaction energy at which the phenol forms hydrogen bonds with HF molecule and Si–OH was calculated to be −0.932 eV. The potential energy was then explored with respect to the attack of the HF molecule to the Si atom in Si–OH. The optimized transition state had an imaginary vibration mode along the direction of the etching reaction. Finally, the stable final and initial state structures along the reaction path from the transition state were determined via intrinsic reaction coordinate (IRC)^45^ calculations. The reaction pathways to the final and initial states were explored by using the IRC calculation along the corresponding imaginary vibration modes and reverse imaginary modes. The geometries of the final steps of the IRC calculations in both directions were used for determining the final and initial states. More details on the IRC calculations are given in ref (46).

## References

[ref1] Laermer F.; Schilp A. Method for Anisotropically Etching Silicon. GE 1992.

[ref2] Laermer F.; Schilp A.; Funk K.; Offenberg M. A. O. M. Bosch Deep Silicon Etching: Improving Uniformity and Etch Rate for Advanced MEMS Applications. In Proc. Technol. Dig. MEMS’99; Florida, USA, 1999; pp 211–216.

[ref3] Li Y.; Zhang H.; Yang R.; Laffitte Y.; Schmill U.; Hu W.; Kaddoura M.; Blondeel E. J.; Cui B. Fabrication of Sharp Silicon Hollow Microneedles by Deep-Reactive Ion Etching towards Minimally Invasive Diagnostics. Microsyst. Nanoeng. 2019, 5, 41 10.1038/s41378-019-0077-y. 31636931 PMC6799813

[ref4] Kamto A.; Liu Y.; Schaper L.; Burkett S. L. Reliability Study of Through-Silicon via (TSV) Copper Filled Interconnects. Thin Solid Films 2009, 518, 1614–1619. 10.1016/j.tsf.2009.07.151.

[ref5] Kim T.; Lee J. Optimization of Deep Reactive Ion Etching for Microscale Silicon Hole Arrays with High Aspect Ratio. Micro Nano Syst. Lett. 2022, 10, 12 10.1186/s40486-022-00155-6.

[ref6] Zhang X.; Zhang Y.; Zhang X.; Guo S. Interface Design and Dielectric Response Behavior of SiO_2_/PB Composites with Low Dielectric Constant and Ultra-Low Dielectric Loss. Surf. Interfaces 2021, 22, 100807 10.1016/j.surfin.2020.100807.

[ref7] Baek G.; Baek J. H.; Kim H. M.; Lee S.; Jin Y.; Park H. S.; Kil D. S.; Kim S.; Park Y.; Park J. S. Atomic Layer Chemical Vapor Deposition of SiO_2_ Thin Films Using a Chlorine-Free Silicon Precursor for 3D NAND Applications. Ceram. Int. 2021, 47, 19036–19042. 10.1016/j.ceramint.2021.03.249.

[ref8] Whitesides G. M. The Origins and the Future of Microfluidics. Nature 2006, 442, 368–373. 10.1038/nature05058. 16871203

[ref9] Hwang J.; Cho Y. H.; Park M. S.; Kim B. H. Microchannel Fabrication on Glass Materials for Microfluidic Devices. Int. J. Precis. Eng. Manuf. 2019, 20, 479–495. 10.1007/s12541-019-00103-2.

[ref10] Park J.-S.; Zhang S.; She A.; Chen W. T.; Lin P.; Yousef K. M.; Cheng J. X.; Capasso F. All-Glass, Large Metalens at Visible Wavelength Using Deep Ultraviolet Projection Lithography. Nano Lett. 2019, 19, 8673–8682. 10.1021/acs.nanolett.9b03333. 31726010

[ref11] Yoo J.-H.; Nguyen H. T.; Ray N. J.; Johnson M. A.; Steele W. A.; Chesser J. M.; Baxamura S. H.; Elhadj S.; McKeown J. T.; Matthews M. J.; Feigenbaum E. Scalable Light-Printing of Substrate-Engraved Free-Form Metasurfaces. ACS Appl. Mater. Interfaces 2019, 11, 22684–22691. 10.1021/acsami.9b07135. 31137930

[ref12] Gaboriau F.; Cartry G.; Peignon M. C.; Cardinaud C. Selective And Deep Plasma Etching of SiO_2_: Comparison between Different Fluorocarbon Gases (CF_4_, C_2_F_6_, CHF_3_) Mixed with CH_4_ or H_2_ and Influence of the Residence Time. J. Vac. Sci. Technol., B: Microelectron. Nanometer Struct.--Process., Meas., Phenom. 2002, 20, 1514–1521. 10.1116/1.1495502.

[ref13] Bliznetsov V.; Lin H. M.; Zhang Y. J.; Johnson D. Deep SiO_2_ Etching with Al and AlN Masks for MEMS Devices. J. Micromech. Microeng. 2015, 25, 087002 10.1088/0960-1317/25/8/087002.

[ref14] Pedersen M.; Huff M. Plasma Etching of Deep High-Aspect Ratio Features Into Fused Silica. J. Microelectromech. Syst. 2017, 26, 448–455. 10.1109/JMEMS.2017.2661959.

[ref15] Holmes P. J.; Snell J. E. A Vapor Etching Technique for the Photolithography of Silicon Dioxide. Microelectron. Reliab. 1966, 5, 337–341. 10.1016/0026-2714(66)90162-4.

[ref16] Jang W. I.; Choi C. A.; Lee M. L.; Jun C. H.; Kim Y. T. Fabrication of MEMS Devices by Using Anhydrous HF Gas-Phase Etching with Alcoholic Vapor. J. Micromech. Microeng. 2002, 12, 297–306. 10.1088/0960-1317/12/3/316.

[ref17] Habuka H. H.; Otsuka T. Reaction of Hydrogen Fluoride Gas at High Temperatures with Silicon Oxide Film and Silicon Surface. Jpn. J. Appl. Phys. 1998, 37, 6123–6127. 10.1143/JJAP.37.6123.

[ref18] Kang J. K.; Musgrave C. B. The Mechanism of HF/H_2_O Chemical Etching of SiO_2_. J. Chem. Phys. 2002, 116, 275 10.1063/1.1420729.

[ref19] Park H.; Antony C. A.; Banerjee J.; Smith J. N.; Agnello G. Prediction of Glassy Silica Etching with Hydrogen Fluoride Gas by Kinetic Monte Carlo Simulations. J. Chem. Phys. 2023, 158, 094709 10.1063/5.0141062. 36889963

[ref20] Huang Z.; Geyer N.; Werner P.; de Boor J.; Gosele U. Metal-Assisted Chemical Etching of Silicon: A Review. Adv. Mater. 2011, 23, 285–308. 10.1002/adma.201001784. 20859941

[ref21] Mikhael B.; Elise B.; Xavier M.; Sebastial S.; Johann M.; Laetitia P. New Silicon Architectures by Gold-Assisted Chemical Etching. ACS Appl. Mater. Interfaces 2011, 3, 3866–3873. 10.1021/am200948p. 21882843

[ref22] Li L.; Liu Y.; Zhao X.; Lin Z.; Wong C.-P. Uniform Vertical Trench Etching on Silicon with High Aspect Ratio by Metal-Assisted Chemical Etching Using Nanoporous Catalysts. ACS Appl. Mater. Interfaces 2014, 6, 575–584. 10.1021/am4046519. 24261312

[ref23] Kong L.; Zhao Y.; Dasgupta B.; Ren Y.; Hippalgaonkar K.; Li X.; Chim W. K.; Chiam S. Y. Minimizing Isolate Catalyst Motion in Metal-Assisted Chemical Etching for Deep Trenching of Silicon Nanohole Array. ACS Appl. Mater. Interfaces 2017, 9, 20981–20990. 10.1021/acsami.7b04565. 28534611

[ref24] Mallavarapu A.; Ajay P.; Barrera C.; Sreenivasan S. V. Ruthenium-Assisted Chemical Etching of Silicon: Enabling CMOS-Compatible 3D Semiconductor Device Nanofabrication. ACS Appl. Mater. Interfaces 2021, 13, 1169–1177. 10.1021/acsami.0c17011. 33348977

[ref25] Bersin R. L.; Reichelderfer R. F. The Dryox Process for Etching Silicon Dioxide. Solid State Technol. 1977, 20, 78–80.

[ref26] Weston D. F.; Mattox R. J. HF Vapor Phase Etching (HF/VPE): Production Viability for Semiconductor Manufacturing and Reaction Model. J. Vac. Sci. Technol. 1980, 17, 466–469. 10.1116/1.570485.

[ref27] Fukasawa T.; Horiike Y. Anisotropic Etching of SiO_2_ Film and Quartz Plate Employing Anhydrous HF. Jpn. J. Appl. Phys. 2003, 42, 4009–4015. 10.1143/JJAP.42.4009.

[ref28] Spierings G. A. C. M. Wet Chemical Etching of Silicate Glasses in Hydrofluoric Acid Based Solutions. J. Mater. Sci. 1993, 28, 6261–6273. 10.1007/BF01352182.

[ref29] Losero E.; Jagannath S.; Pezzoli M.; Goblot V.; Babashah H.; Lashuel H. A.; Galland C.; Quack N. Neuronal Growth on High-Aspect-Ratio Diamond Nanopillar Arrays for Biosensing Applications. Sci. Rep. 2023, 13, 5909 10.1038/s41598-023-32235-x. 37041255 PMC10090193

[ref30] Linklater D. P.; Baulin V. A.; Juodkazis S.; Crawford R. J.; Stoodley P.; Ivanova E. P. Mechano-Bactericidal Actions of Nanostructured Surfaces. Nat. Rev. Microbiol. 2021, 19, 8–22. 10.1038/s41579-020-0414-z. 32807981

[ref31] Yoon S.-S.; Khang D.-Y. Vertical Arrays of SiO_2_ Micro/Nanotubes Templated from Si Pillars by Chemical Oxidation for High Loading Capacity Buoyant Aquatic Devices. ACS Appl. Mater. Interfaces 2013, 5, 13441–13447. 10.1021/am4043504. 24313459

[ref32] Tulli D.; Hart S. D.; Mazumder P.; Carrilero A.; Tian L.; Koch K. W.; Yongsunthon R.; Piech G. A.; Pruneri V. Monolithically Integrated Micro- and Nanostructured Glass Surface with Antiglare, Antireflection, and Superhydrophobic Properties. ACS Appl. Mater. Interfaces 2014, 6, 11198–11203. 10.1021/am5013062. 24960031

[ref33] Lee H. J.; Tak H. W.; Kim S. B.; Kim S. K.; Park T. H.; Kim J. Y.; Sung D.; Lee W.; Lee S. B.; Kim K.; Cho B. O.; Kim Y. L.; Lee K. C.; Kim D. W.; Yeom G. Y. Characteristics of High Aspect Ratio SiO_2_ Etching using C_4_H_2_F_6_ Isomers. Appl. Surf. Sci. 2023, 639, 158190 10.1016/j.apsusc.2023.158190.

[ref34] Tak H. W.; Lee H. J.; Wen L.; Kang B. J.; Sung D.; Bae J. W.; Kim D. W.; Lee W.; Lee S. B.; Kim K.; Cho B. O.; Kim Y. L.; Song H. D.; Yeom G. Y. Effect of Hydrofluorocarbon Structure of C_3_H_2_F_6_ Isomers on High Aspect Ratio Etching of Silicon Oxide. Appl. Surf. Sci. 2022, 600, 154050 10.1016/j.apsusc.2022.154050.

[ref35] Huang S.; Huard C.; Shim S.; Nam S. K.; Song I.-C.; Lu S.; Kushner M. J. Plasma Etching of High Aspect Ratio Features in SiO_2_ using Ar/C_4_F_8_/O_2_ Mixtures: A Computational Investigation. J. Vac. Sci. Technol., A 2019, 37, 031304 10.1116/1.5090606.

[ref36] Chang C.; Wang Y.-F.; Kanamori Y.; Shih J.-J.; Kawai Y.; Lee C.-K.; Wu K.-C.; Esashi M. Etching Submicrometer Trenches by using the Bosch Process and its Application to the Fabrication of Antireflection Structures. J. Micromech. Microeng. 2005, 15, 580 10.1088/0960-1317/15/3/020.

[ref37] Hoshino T.; Nishioka Y. Etching Process of SiO_2_ by HF Molecules. J. Chem. Phys. 1999, 111, 2109 10.1063/1.479480.

[ref38] Chowdhury T.; Hidayat R.; Mayangsari T. R.; Gu J.; Kim H.-L.; Jung J.; Lee W.-J. Density Functional Theory Study on the Fluorination Reactions of Silicon and Silicon Dioxide Surfaces using Different Fluorine-Containing Molecules. J. Vac. Sci. Technol., A 2019, 37, 021001 10.1116/1.5081490.

[ref39] Hidayat R.; Kim H.-L.; Khumaini K.; Chowdhury T.; Mayangsari T. R.; Cho B.; Park S.; Lee W.-J. Selective Etching Mechanism of Silicon Oxide Against Silicon by Hydrogen Fluoride: A Density Functional Theory Study. Phys. Chem. Chem. Phys. 2023, 25, 3890 10.1039/D2CP05456F. 36647706

[ref40] Kim D. H.; Kwak S. J.; Jeong J. H.; Yoo S.; Nam S. K.; Kim Y. J.; Lee W. B. Molecular Dynamics Simulation of Silicon Dioxide Etching by Hydrogen Fluoride Using the Reactive Force Field. ACS Omega 2021, 6, 16009–16015. 10.1021/acsomega.1c01824. 34179646 PMC8223409

[ref41] Becke A. D. Density-Functional Thermochemistry. III. The Role of Exact Exchange. J. Chem. Phys. 1993, 98, 5648–5652. 10.1063/1.464913.

[ref42] Lee C.; Yang W.; Parr R. G. Development of the Colle-Salvetti Correlation-Energy Formula into a Functional of the Electron Density. Phys. Rev. B 1988, 37, 785 10.1103/PhysRevB.37.785. 9944570

[ref43] Frisch M. J.; Trucks G. W.; Schlegel H. B.; Scuseria G. E.; Robb M. A.; Cheeseman J. R.; Scalmani G.; Barone V.; Petersson G. A.; Nakatsuji H.; Li X. Gaussian 16. Revision C.01, Gaussian, Inc.: Wallingford, CT, 2016.

[ref44] The convergence criteria for geometric optimizations are as follows: the energy change per step of 1.0 × 10^–6^ hartree, the maximum force of 3.0 × 10^–4^ hartree/Bohr, the root-mean-square (RMS) values of all forces of 1.0 × 10^–4^ hartree/Bohr, the maximum atomic coordinate per step of 1.2 × 10^–3^ Bohr, and the RMS values of all atomic coordinates per step of 6.0 × 10^–4^ Bohr

[ref45] Gonzalez C.; Schlegel H. B. An Improved Algorithm for Reaction Path Following. J. Chem. Phys. 1989, 90, 2154–2161. 10.1063/1.456010.

[ref46] The IRC calculations were performed are as follows: A force constant for the calculation was determined in the initial step. A step size was 0.1 (amu)^1/2^Bohr and the maximum numbers of calculation times was 200

